# From Personalized Medicine to Personalized Aging Services

**DOI:** 10.1093/geroni/igab047

**Published:** 2021-10-25

**Authors:** Allen Glicksman, Misha Rodriguez, Lauren Ring, Michael Liebman

**Affiliations:** 1 Department of Family and Community Health, School of Nursing, University of Pennsylvania, Philadelphia, Pennsylvania, USA; 2 Asociación Puertorriqueños en Marcha, Philadelphia, Pennsylvania, USA; 3 Philadelphia, Pennsylvania, USA; 4 IPQ Analytics, Kennett Square, Pennsylvania, USA

**Keywords:** Immigrant, LEP (Limited English Proficiency), Medical model, Managed care

## Abstract

As medical models become more ubiquitous in developing strategies to provide long-term care services and support (LTSS), we need to ask whether these models adequately account for sources of diversity and disadvantage that affect access to and use of services by older adults. Medical models typically focus on categorizing information about the individual in order to clearly define current health status and appropriate treatment. Any individual, however, reflects the sum of their life experiences. Therefore, this medicalization approach can miss key factors in determining health outcomes including social determinants of health. Just as importantly, this approach can miss issues of values, beliefs, and assumptions that older adults can bring into the encounter with service providers. This issue is especially important when dealing with older migrant communities. Beliefs and attitudes shaped in their place of origin, as well as the migration experience, can influence levels of trust and resulting decisions regarding the use of LTSS. We need to integrate an understanding of how these beliefs and attitudes affect decision making into any model designed to improve the lives of older persons.

**Translational Significance:** To improve access and use of services by older adults it is critical to understand both their status as well the social and cultural contexts that shape their worldview. This is especially important when dealing with immigrant communities whose attitudes can be shaped by their experience in their native land and by the process of immigration and adoption of their new environment.

Aging, uniquely, involves processes that are shared among all living beings and that effectively begin at birth. However, even among humans, aging is not necessarily experienced or perceived identically across disparate population groups, for example, gender, race, and nationality. As a result, organizations focused on providing long-term social services and supports (LTSS) need to understand the sources and extent of these differences to optimize the *how*, *where*, *when*, and *what* that they offer. Personal or “precision” medicine provides a model for such targeting but may not be the best approach for LTSS because it fails to recognize fully the role of social determinants, an understanding of which is critical for effective delivery of aging services. Medicalization of LTSS, that is, linking LTSS to health care delivery in current Medicaid-managed long-term care initiatives, must pay greater attention to these social determinants, and as appropriate, also cultural differences and factors affecting trust.

## Limitations of the Medical Model for LTSS

The current emphasis on medicalization of LTSS services must be responsive to the role such disparities play in health outcomes for older adults. Concerns have been raised since the 1990s about the possible negative impact of medicalization on the lives of older persons receiving LTSS. This reflects medicine’s emphasis on therapeutic intervention as opposed to prevention. Targeted improvements in the overall management of aging can yield significantly better outcomes for individuals and agencies providing services (Bierman & Tinetti 2016). This requires a comprehensive evaluation of these population groups, extending beyond health, to include environment, food, housing, transportation, and social connectedness, elements of resiliency, and both family and community support. Providers face challenges carrying out this evaluation within a medical model.

Supporters of the medically oriented approach to aging services argue that this will improve health, decrease cost, and provide more personalized care for older persons in need of social and health services. However, health disparities associated with poverty, race, sex, gender identity, or other sociodemographic characteristics challenge such a person-centered approach. Much debate about medicalization of aging services focuses on whether it will enhance or impede the formal care system from achieving the goal of reducing or eliminating these disparities.

In its favor, medicalized LTSS seeks to categorize and classify sources of diversity linked to disadvantage in its use of the International Statistical Classification of Diseases and Related Health Problems (ICD-10), Diagnostic and Statistical Manual of Mental Disorders, and International Classification of Functioning, Disability and Health (American Psychiatric Association 2013; World Health Organization 1992, 2001). Identifying the key elements defining health disparities and collecting information on these elements should lead to “personalizing” service to disadvantaged groups. However, these classification systems are not free of bias. Studies suggest important group-based differences in the performance of biometric instruments (pulse oximeters and sensitivity to skin color), the effect of measurement context (blood pressure assessment), and reference values for physiologic parameters (glomerular filtration rates differ among races; Feiner et al. 2007). The new ICD-10 Z codes attempt to capture social needs, such as inadequate housing or food insecurity, but these likely do not fully capture the full effects of social determinants nor do they address critical cultural differences.

## Taking Social Determinants Seriously in LTSS: Culture

The true complexity of the real world does not end there.

Appropriate alignment of LTSS with older patients requires consideration of their culture and life experiences. Culture and life experience may also influence their own sense of need and desire to report that need. Similarly, trust in medical providers may reflect a group’s history of interaction with authority, medical, or otherwise—“trust determinants.” Therefore, health and social service systems need to focus on two challenges that could allow an older person to take advantage of the services offered.

The first is to remove barriers to appropriate programs and services. Some of these barriers are cultural. Therefore, the perspective of the older adult must be understood in order to identify and fully understand such barriers.

The second goal is to communicate the importance of health maintenance activities in culturally appropriate forms—for example, language/dialect of the older adult (and/or family), with sensitivity to cultural differences in examples used and between both provider and client.

Once such communication channels are established, they must be maintained and updated to adapt to changes at the individual, family, and community levels, and incorporated into the design and implementation of services.

It is critical to understand and include the personal experience, assumptions, and beliefs of the older adult as they affect their interactions with the formal care systems. The social determinants of health (SDoH) are potentially modifiable, with enough resources and commitment. Cultural and trust determinants of health, however, are engrained and difficult to modify. These attitudes and beliefs will shape the decision to use LTSS services and affect the accessing and use of those services.

## A Key Social Determinant for LTSS: Migration Status

Nowhere are these social and cultural determinants more important than when dealing with older adults who are migrants. Migration status has often been ignored as a source of diversity because of the focus placed on conventional racial/ethnic categories mandated by the federal government. Migration has been typically buried. Minority status (broadly racial/ethnic categories, e.g., Black non-Hispanic, Asian) and primary language are usually the only data collected to identify persons who migrated to the continental United States. This is curious because, in 2018, approximately 52.5 million people in the United States were 65 years and older and of these, 7.3 million (13.9 %) were foreign-born. Between 2018 and 2060, the older, foreign-born population is expected to increase by about 200% to 22.0 million (Mizoguchi et al., 2019).

The value norms and assumptions regarding the need and use of LTSS services reflect three characteristics of the older immigrant—circumstances of origin, circumstances of migration, and circumstances of current residence. These experiences affect the level of trust the older person and family have in the LTSS system. Trust integrates reason, emotion, and experience.

### Circumstances of Origin

For persons migrating to mainland United States (including Puerto Rico), norms and values, social structure, family life, and political environment, as well as the physical environment in which they grew up, can continue to shape their worldview even as they enter a new country. What role did family and government play in planning and programming support services in their place of origin? What happens through migration if extended family becomes scattered or unable to support one another as they would have “back home”? The very different structures of health and LTSS systems—if one existed—are their first experience with these services. In addition, the person may have always been a member of a minority or disadvantaged group (rural, poor, etc.) without access to an LTSS system. Attitudes here can shape later experiences. For example, persons born in rural China may have had a vastly different health care experience than those born in large cities; these further differ across north–south boundaries. Finally, the role and trust in the political system can shape attitudes toward any large system, government or not. In Puerto Rico, governmental misuse of medical aid funds and historically complicated and splintered relationships with the United States government can lead to distrust concerning access to and availability of benefits and support services.

### Circumstances of Migration

Migrants rarely represent the diversity of people from a single place of origin. Rather, persons who migrate usually represent specific strata within the society or have specific status that either encourages or forces migration. In some cases, such as fleeing persecution, the “pull” element can be defined by whatever country will accommodate refugees. Therefore, specifics of migration, including age at migration, forced/voluntary migration, and solo versus family migration (or migration to be with family) must be considered.

A good example is the migration from the former Soviet Union to the United States in the 1990s. Although some arrived as refugees (based on persecution under the Soviet regime), many arrived because of the nuclear disaster at Chernobyl in 1986. Fear that their grandchildren could develop untreatable cancers spurred some parents’ decisions. Often parents, who had only one child, felt compelled to migrate with as many of their family as possible. This internal pressure, coupled with a total lack of trust in the system, that is, dishonesty about cancer risk, shaped their worldview in the United States as well as relations among family members (Glicksman, 2017).

It is also important to determine whether the migrant considers the migration as permanent or temporary, especially when migration is politically forced or due to a natural disaster. Some migrants may initially believe their migration is only temporary, which affects investment in learning new socioenvironmental and political-institutional environments.

### Circumstances of Current Residence

What is the legal status of the migrant in the United States and how does that affect access to services? The professional community in aging and health services assigns ethnicity labels to categorize individuals for reports on sociodemographic characteristics and to make decisions about interventions. For example, when categorized in most health and social service systems, Puerto Ricans, Dominicans, Mexicans, and so forth, all become “Hispanic,” creating categorical unity and “othering” through language. Chinese migrants, rural or urban, level of education, and so forth; mainland or Hong Kong—all become “Chinese.” Often, regional dialects spoken by older adults or national language differences (Cantonese or Mandarin) are not recognized.

This means that the older Spanish or Chinese speaker, no matter what their status or place of origin, now must become part of a “community” of persons based on seemingly common language alone. That person is now reclassified either by language or region of the world, for example, “Africa.” For example, Chinese is both a national origin and a language. Nonethnic Chinese who speak Mandarin become “Chinese” in some agency categorizations. Reclassification plays a significant role in the way the older adult is “assigned” or expected to seek services from agencies serving that language group.

Also, the person may not have been a member of a minority group in their place of origin but became one here in the United States. The reframing as a minority group member in the American classification affects services offered to the older adult. It can also shape how the older adult views formal systems and the trust—or lack—that they have in those formal systems. Assignment to one ethnicity rather than another (e.g., Puerto Rican vs. another Spanish-speaking group) may introduce different treatments and encourage disparities. Migrants may also have experienced unemployment, and change in class/economic standing, depending on migration experience.

## Trust and Targeting of LTSS

These circumstances play critical roles in the shaping of perceptions, values, and, most of all, trust in LTSS and health systems and are critical in the development of trust networks—that is, networks within migrant communities where information is shared and seen as trustworthy by most members. Sometimes this may involve a trusted doctor or case manager. Often, trust networks are informal and exist among the migrant populations themselves, independent of the agencies assigned to serve them. Depending on culture and place of origin, these networks may reflect family ties or shared specific immigration experience (cohort, specific place of origin, activity, such as attending a recreation center program, religion/spirituality, etc.; Ring et al., 2020).

The presence or absence of trust will influence the decision whether or not to use services. Only when a crisis arises and there is no alternative to intervention by formal providers does trust becomes a reduced component in decision making. Can these circumstances be classified, measured, and scored to help understand how an older person might respond to the question of need of LTSS services? If trust networks work differently among different migrant groups, how do we account for this in data?

More critically, if trust is so central to the interaction of the older adult and the formal system, how do we know that the older person and their families are being truthful and open with the person asking the questions? And how do we know the older adult (or family member) is providing accurate information? Can lack of trust in providers lead older persons to respond with answers to questions they believe will trigger the services they desire? Can “managing” the managers, that is, representatives of the formal system, be a sign of resilience in their effort to retain control over their own lives, and if so, how is that handled? If they do provide such answers, is that misuse or a sign of resilience when trying to manage a system that is assumed to be unsympathetic to the older adult?

Beyond this, we also need to understand the role of family throughout this process. What data are needed to understand the role family plays at all three levels—origin, immigration experience, and relocation? More importantly, can circumstance predict values or only help understand the roots of these values? If there is basic distrust, then will educational programs about specific interventions work? What health system expectations and assumptions exist and where did they originate? Was there a social service system in place of origin and how did that work?

In all cases, the goal of minimization/elimination of health disparities requires understanding the worldview of the persons being served. In vaccine hesitancy, U.S.-born persons can hold beliefs that preclude following public health initiatives. The experience of migrants is a window into a larger set of questions about the adequacy of the medicalization approach because of the assumptions built into that system. We need to understand the process of decision making with respect to LTSS, which often involves group and community factors, and not just certain characteristics of the person—and in some cases also the family members—making the decision.

## The Role of Resilience

Understanding the impact of migration on the health and health behaviors of older adults is not limited to negative aspects of their life experiences. Many of these immigrants have survived to old age because of their own resilience and the support of their families and communities both in their place of origin and their new homes. The very act of building new communities in the United States is in itself an important sign of the strength and abilities of the members of these communities. Health and social service agencies sometimes only see these community efforts as sources of access to community members without considering the significance that they exist at all. The sense of self-efficacy among some of these older adults is at the heart of their belief that they alone and not family members or agency staff know what is best for them and that must be taken into account when working with older immigrants as well. Finally, information can flow within these communities that is considered more reliable than information from outside sources because of a sense of shared experience and common values. This can lead to more positive outcomes but it can also lead, as mentioned above, to erroneous information being shared and, at times, suggestions on how to better manage the managers in health and social services. Real resilience exists among many older immigrants. Without the understanding of how communities work, how reliable information may be shared, and the sources of that resilience, it will be very difficult to effectively serve these older persons.

## Modeling the Interactions Between Older Immigrants and the Health and Social Care Systems

**Figure 1.**
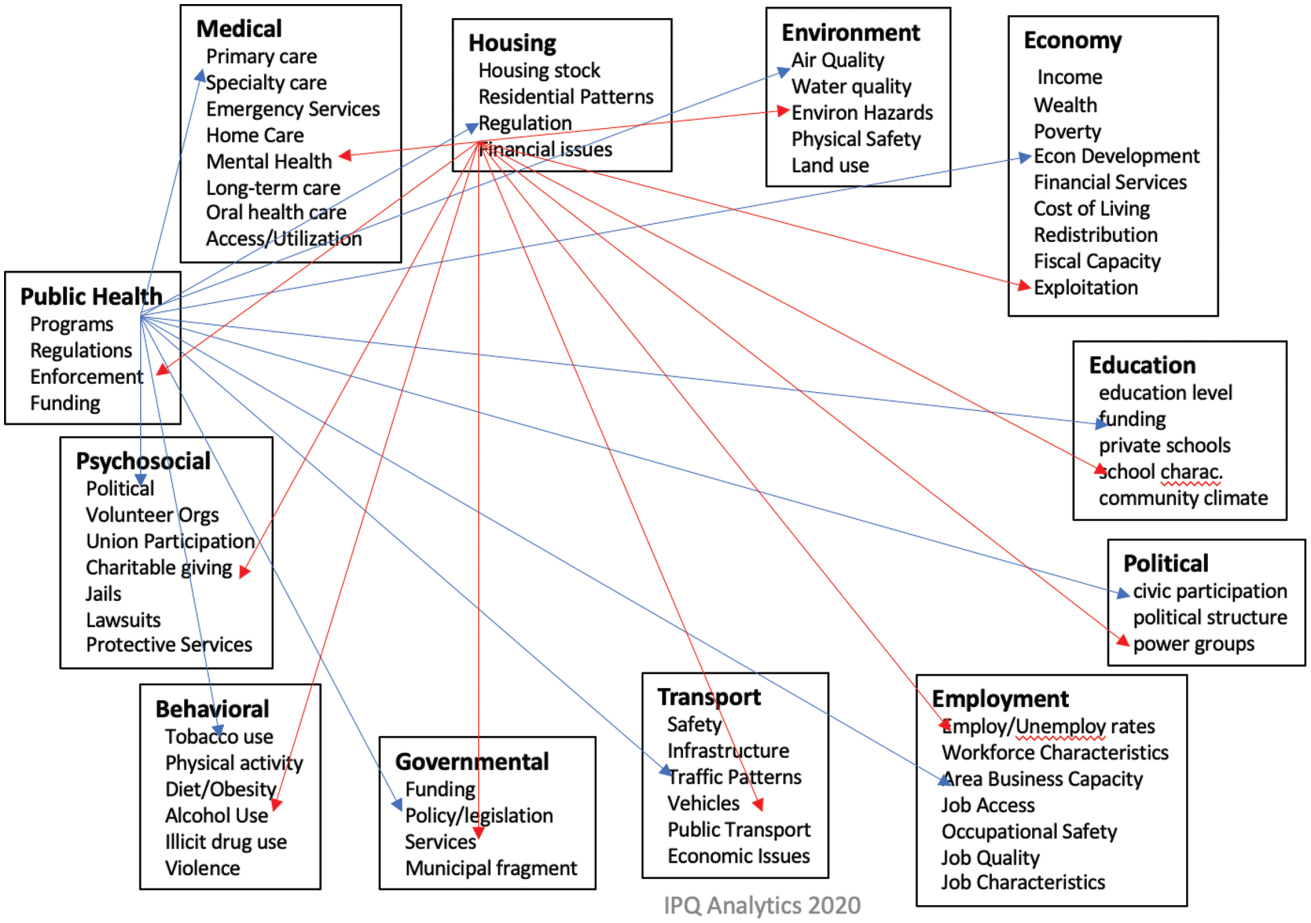
Model of interactions among social determinants of health.

The client populations for these services are best characterized by “real-world factors” that are both complicated and complex. This reality can present significant challenges to approaches based on medicalization, in which assignment of characteristics to predefined “diagnoses” is fundamental but can be limiting. The social interaction model (SIM) we are developing addresses this challenge by implementing a unique knowledge graph (KG) that supports a continuous learning system. The SIM-KG is constructed using concepts from experts and published literature and creates a network that is fully connected, that is, every concept is potentially connected directly or indirectly to every other concept. We initiated this in the SIM-KG using the Centers for Disease Control and Prevention’s definition of the social determinants of health that, at the top level, include medical, housing, environment, economy, education, political, employment, transport, governmental, behavioral, psychosocial, and public health. Each of these has multiple sublevels (e.g., environment) including air quality, water quality, environmental hazards, physical safety, and land use. As exemplified in Figure 1, the potential connectivity of this graph grows as a function of *n*(*n* − 1)/2, where *n* is the number of concepts.

This form of representation can be readily extended as we are doing through the addition of cultural and trust features, which are then directly linked into these SDoH and which can be significant in identifying critical priorities, and so forth in distinct population groups.

The actual relationships, that is, links between concepts, can be used to (a) codify existing knowledge from experts or publications; (b) create questionnaires/surveys to be used to analyze client populations, especially of diverse backgrounds; (c) identify gaps in existing linkages between concepts that might be important in certain populations; and (d) identify conflicting perceptions among diverse populations that could affect acceptance of specific services.

By example, offering housing that inadequately considers the family structure within a specific culture can, and has, led to expensive investment of resources with less than positive results.

## Transforming Managed Care

How would this approach be implemented and how could that provide better outcomes than those based on the medical model?

As a start, trust needs to be built through communication and community building. Simply developing a Spanish or Chinese language version of a standard questionnaire will not address these key issues. The medical community must work harder to identify and work with key community stakeholders and to listen to their needs and ideas. We must prioritize localized outreach and programming that builds on the strength of migrant community leaders.

In addition, relationship building is a continuous process, not just in times of crisis. For example, as we have seen with vaccine hesitancy, translating messages into Spanish will not erase years of distrust and neglect within the Puerto Rican diaspora. Even a common religion may not be adequate to overcome cultural differences, as evidenced by some Catholic bishops who oppose the Vatican’s position on vaccination against coronavirus disease 2019. If we do not find ways to integrate attitudes and beliefs into models, then the postdisruption world will not look so different from the predisruption one.

To facilitate the collection of critical data to carry out the “personalizations” noted above, we have been developing models of the interactions between an individual and their family and community, both pre- and postmigration, and utilizing that as the basis to develop surveys. This enables direct comparisons within and across communities as a means for understanding how to prioritize potential services and communication channels necessary to facilitate the interactions with individuals and communities.

The medicalization of aging services will likely continue, but we should also remember challenges and limitations that exist in how medicine is currently practiced. Ideally, the expanded approach described above and the models being built will enable a progressive shift from one of “therapeutics” to one of “prevention” or at least to a much earlier recognition of the prioritization of services that more closely address the particular needs of individuals and communities; not a “one size fits all” approach to social services and to medicine.

The key to fully applying this approach is threefold. First, existing models of care that integrate a “case management” approach must be part of any system that offers care. A case manager can ask the key questions about the life experience and current status of the older adult that will allow for realistic future planning. Expecting a person to simply adhere to whatever is prescribed without considering whether the person is even inclined to listen to the recommendations is simply not enough. To support that, the second element is to add more items to the list of questions asked of each person, beginning with their place of origin, number of years living in the United States, and so on. Third, the health care and social service agencies that provide care must partner with community organizations that are known to and trusted by members of the immigrant group being served. These must be partnerships based on equal respect and sharing of resources, not a top-down model. These community groups understand issues facing the older adults that may not appear on a list of standard intake questions but that are critical for effectively serving the community. In doing so, older persons can be encouraged to begin using services at a lower level of acuity or distress, so that they do not only become known to the formal system before a moment of crisis (e.g., roof collapse, medical emergency) when less can be done to change the outcome and when costs are highest.

The suggestions we are making will have a marked, positive effect on the health and well-being of older immigrants. However, all these suggestions will benefit all older persons, including those who were born in the United States. While much of the promise of managed care is based on the commitment to “personalized service,” such a commitment can only be honored if the whole person, not only their current health status and sociodemographic characteristics, are taken into account. Finding out through a case manager that an older person has never seen a physician and is concerned about doing so now can be a critical piece of information that shapes the way in which services are offered. Databases restricted to close-ended questions that predefine all possible answers do not allow for these special cases that must be understood if they are to receive appropriate treatment.

Managed care systems must go beyond current medicalized models of care and integrate not only the social determinants of health but the whole person—including background, culture, and life experience—to fully serve the coming generation of older persons.
